# Cardiovascular risk factors and mortality in hospitalized patients with COVID-19: systematic review and meta-analysis of 45 studies and 18,300 patients

**DOI:** 10.1186/s12872-020-01816-3

**Published:** 2021-01-07

**Authors:** Angelo Silverio, Marco Di Maio, Rodolfo Citro, Luca Esposito, Giuseppe Iuliano, Michele Bellino, Cesare Baldi, Giuseppe De Luca, Michele Ciccarelli, Carmine Vecchione, Gennaro Galasso

**Affiliations:** 1 Department of Medicine, Surgery and Dentistry, University of Salerno, Baronissi, Salerno Italy; 2 Division of Cardiology, Cardiovascular and Thoracic Department, University Hospital ‘‘San Giovanni di Dio e Ruggi d’Aragona’’, Salerno, Italy; 3 Division of Cardiology, Azienda Ospedaliera-Universitaria ‘‘Maggiore della Carità’’, Eastern Piedmont University, Novara, Italy; 4 Vascular Pathophysiology Unit, IRCCS Neuromed, Pozzilli, Isernia Italy

**Keywords:** Novel coronavirus, SARS-CoV-2, COVID-19, Cardiovascular risk factors, Hypertension, Smoking, Diabetes, Mortality, Outcome

## Abstract

**Background:**

A high prevalence of cardiovascular risk factors including age, male sex, hypertension, diabetes, and tobacco use, has been reported in patients with Coronavirus disease 2019 (COVID-19) who experienced adverse outcome. The aim of this study was to investigate the relationship between cardiovascular risk factors and in-hospital mortality in patients with COVID-19.

**Methods:**

MEDLINE, Cochrane, Web of Sciences, and SCOPUS were searched for retrospective or prospective observational studies reporting data on cardiovascular risk factors and in-hospital mortality in patients with COVID-19.

Univariable and multivariable age-adjusted analyses were conducted to evaluate the association between cardiovascular risk factors and the occurrence of in-hospital death.

**Results:**

The analysis included 45 studies enrolling 18,300 patients. The pooled estimate of in-hospital mortality was 12% (95% CI 9–15%). The univariable meta-regression analysis showed a significant association between age (coefficient: 1.06; 95% CI 1.04–1.09; p < 0.001), diabetes (coefficient: 1.04; 95% CI 1.02–1.07; p < 0.001) and hypertension (coefficient: 1.01; 95% CI 1.01–1.03; p = 0.013) with in-hospital death. Male sex and smoking did not significantly affect mortality. At multivariable age-adjusted meta-regression analysis, diabetes was significantly associated with in-hospital mortality (coefficient: 1.02; 95% CI 1.01–1.05; p = 0.043); conversely, hypertension was no longer significant after adjustment for age (coefficient: 1.00; 95% CI 0.99–1.01; p = 0.820). A significant association between age and in-hospital mortality was confirmed in all multivariable models.

**Conclusions:**

This meta-analysis suggests that older age and diabetes are associated with higher risk of in-hospital mortality in patients infected by SARS-CoV-2. Conversely, male sex, hypertension, and smoking did not independently correlate with fatal outcome.

## Introduction

Coronavirus disease 2019 (COVID-19) is a recently recognized infective disease caused by a new betacoronavirus, which has sparked in Hubei province, China, and has spread rapidly worldwide taking on pandemic proportions. [1] Owing to the frequent involvement of respiratory tract in humans, the viral agent is also known as severe acute respiratory syndrome coronavirus 2 (SARS-CoV-2). [2]

Up to date, COVID-19 has affected over 7 millions of people and has been associated with more than 400 thousand deaths worldwide. [3] A large discrepancy in the rates of mortality has emerged across reports, resulting in an open debate involving healthcare administrators, physicians and researchers in several countries. Beyond the strategies adopted by governments and health-care resources availability, [4] the inconsistency of overall case-fatality rate between centers seems largely influenced by the clinical profile of the patients enrolled. [5, 6]

Previous studies have investigated the association between baseline characteristics and outcome of patients with COVID-19, and have showed that age and multiple comorbidities may precipitate clinical course during hospitalization. [5, 7, 8] Cardiac injury, defined by increased serum troponin levels, emerged as an independent predictor of mortality in COVID-19, particularly when associated to underlying cardiovascular disease. [9] Moreover, traditional cardiovascular risk factors including age, hypertension, diabetes and smoking, were frequently reported in critically ill cases and seemed to affect in-hospital outcome. [9–11] These conditions are highly prevalent in high-income Asian and Western countries and represent a matter of concern, especially considering population growth and ageing. [12].

Whether cardiovascular risk factors may play a role on clinical course and outcome of patient infected by SARS-CoV-2 remains unclear. A quantitative synthesis of observational data may help to understand the effect of cardiovascular risk factors on the outcome of patients hospitalized for COVID-19, and to identify parameters potentially useful for prognostic stratification.

The aim of this meta-analysis was to investigate the burden of cardiovascular risk factors and the rate of fatal outcome in patients infected by SARS-CoV-2, and to explore their relationship during hospitalization.

## Methods

This study was designed according to the Meta-analysis of Observational Studies in Epidemiology (MOOSE) statement. [13] The review protocol was not registered on PROSPERO.

### Data sources and searches

A comprehensive MEDLINE, Cochrane, Web of Science, and SCOPUS literature search was performed until April 27, 2020. Studies dealing with the clinical characteristics and outcome of patients hospitalized for COVID-19, including those presented or published in other languages, were selected. Articles in languages other than English were screened by using on-line translators and through contacts with researchers from other countries. The following search strategies were used: (1) MEDLINE—(“COVID-19”[All Fields] OR “COVID 19”[All Fields] OR “severe acute respiratory syndrome coronavirus 2”[All Fields] OR “2019-nCoV”[All Fields] OR “SARS-CoV-2”[All Fields]) AND (“risk factors”[MeSH terms] OR “hypertension”[All Fields] OR “smoking”[All Fields] OR “age”[All Fields] OR “diabetes”[All Fields] OR “sex”[All Fields] OR “gender”[All Fields] OR “comorbidities”[All Fields] OR “cardiovascular disease”[All Fields] OR “outcome”[All Fields] OR “mortality”[All Fields]); Cochrane Library, Web of Science and SCOPUS—“COVID-19” OR “COVID 19” OR “SARS-CoV-2” OR “severe acute respiratory syndrome coronavirus 2” OR “2019-nCoV”. Search was conducted by using the Thomson Reuters EndNote X7 software.

### Study selection

Citations were screened on the title and abstract level by three independent reviewers (A.S., L.E. and G.I.), and potentially eligible reports were retrieved and scrutinized in full text. Divergences were resolved by discussion and consultation with a fourth investigator (G.G.). The full-size articles published in peer-reviewed journals were considered for this meta-analysis.

Prospective and retrospective observational studies were included if they met the following pre-specified criteria: (I) inclusion of patients hospitalized for COVID-19; (II) data on in-hospital mortality; (III) data on baseline cardiovascular risk factors. Studies reporting only surrogate outcome measures or with largely incomplete data on in-hospital outcome were excluded. Studies reporting data on special populations (e.g. pregnant women, children) were excluded. For papers collecting overlapping data, only studies with the largest number of patients were selected. In some doubtful cases, corresponding authors were contacted for requests for clarifications.

### Data extraction and quality assessment

Two investigators (A.S. and L.E.) independently extracted data by using an agreed predefined spreadsheets reporting first author, journal, year of publication, study design, region and country, period of enrollment, sample size, in-hospital mortality, demographic and clinical cardiovascular risk factors (age, male gender, hypertension, diabetes and smoking). Data on the severity of the disease expressed by the percentage of acute respiratory distress syndrome (ARDS) and of invasive mechanical ventilation (IMV) use, as well as data on clinical setting expressed by the percentage of admission in intensive care unit (ICU) were collected. Disagreements were resolved by consensus among all the investigators.

Two unmasked reviewers (L.E., G.I.) evaluated the quality of the studies on pre-specified electronic forms according to the Newcastle–Ottawa scale items. The reviewers independently appraised study selection, comparability, and outcome of each report, and divergences were resolved after consensus.

### Study outcome

The study outcome measure was the occurrence of death during hospitalization.

### Data synthesis and analysis

The meta-analysis was performed by estimating the mean in-hospital mortality over all studies using random effects models with restricted maximum-likelihood estimator. Random-effects model was preferred for estimating the average effect and its precision, which would give a more conservative estimate of the 95% confidence interval (CI), due to the heterogeneity within and between studies. Studies with larger sample size and therefore a smaller standard error received more weight when calculating the mean survival proportions. The individual study proportions of outcomes were converted using the Freeman–Tukey double arcsine transformation method before the pooled analysis. The summarized proportions in the original scales were calculated as the back-transformation of the arcsine transformed estimates. Raw data on the prevalence of cardiovascular risk factors were collected for each study included in this meta-analysis.

Multiple univariable meta-regression analyses were performed to appraise the possible association between the proportion of single cardiovascular risk factors and the risk of death, as well as the degree of heterogeneity among studies. In order to assess whether the associations resulted at univariable analyses were age-independent, data on each cardiovascular risk factor were entered in age-adjusted multivariable meta-regression models.

These analyses were performed using the logarithmic transformation of the proportions, then back transformed through the exponential function.

To appraise for the influence of clinical setting on the study outcome, we conducted a subgroup analysis for studies including only ICU patients versus studies enrolling mixed population (both ward and ICU) and tested any interaction between subgroups.

The hypothesis of statistical heterogeneity was tested by means of Cochran Q statistic and the null hypothesis of statistical homogeneity was refused if p values were less than 0.10. I [2] values < 40%, 40–60% and > 60% indicated low, moderate, and substantial statistical inconsistency, respectively. [14] Funnel plots for the primary outcome were used to evaluate the presence of publication bias, heterogeneity of studies, or data irregularities. The significance of asymmetry was explored using visual inspection tested by a rank correlation test based on Kendall’s τ. All analyses were performed using R version 3.5.1 (R Foundation for Statistical Computing, Vienna, Austria).

## Results

**Fig. 1:**
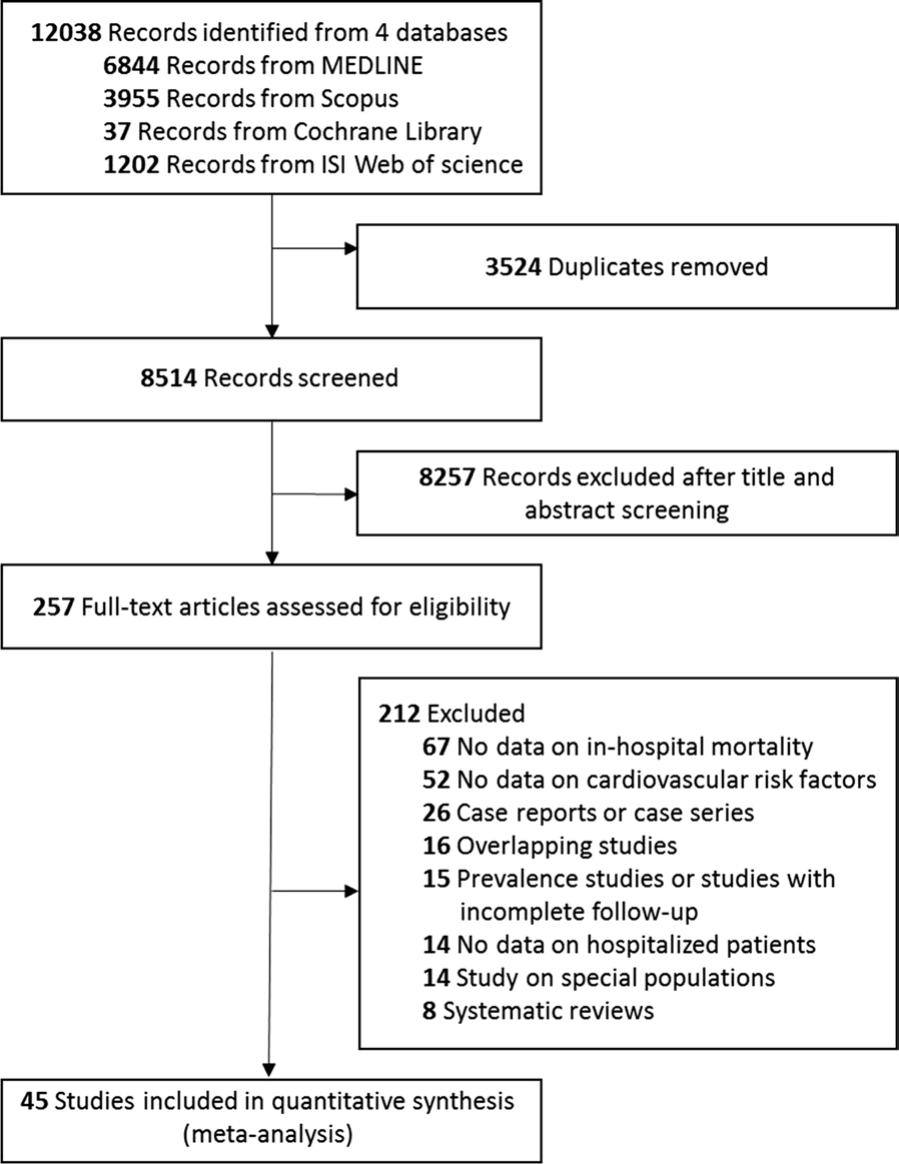
Flow diagram of the study selection process

Of 12,038 reports initially identified, we retrieved 8,514 studies through merging of data from independent searches and removing duplicates. Sixteen studies have been excluded because of overlapping populations. During screening and eligibility assessment, we identified 45 full-size articles enrolling 18,300 patients. [7–11, 15–54] The baseline features of the study populations, where available, are reported in Table 1 and Additional file 1. The study selection process is depicted in Fig. 1.

**Table 1:** Studies included in the meta-analysis

Author	Journal	Region, country	Time of enrollment	End of follow-up	Patients, N
Arentz M	JAMA	Washington State, US	From February 20, 2020 to March 5, 2020	March 17, 2020	21
Barrasa H	Am J Respir Crit Care Med	Basque country, Spain	From March 4, 2020 to March 31, 2020	March 31, 2020	48
Bhatraju K	N Eng J Med	Washington State, US	From December 24, 2019 to March 9, 2020	March 23, 2020	24
Cai Q	Allergy	Guangdong, China	From January 11, 2020 to February 6, 2020	March 6, 2020	298
Chen G	J Clin Invest	Hubei, China	From December 30, 2019 to January 27, 2020	February 2, 2020	21
Chen N	Lancet	Hubei, China	From January 1, 2020 to January 20, 2020	January 25, 2020	99
Cheng Y	Kidney Int	Hubei, China	From January 28, 2020 to February 11, 2020	February 29, 2020	701
Cui J	J Thromb Haemost	Hubei, China	From January 30, 2020 to March 22, 2020	March 22, 2020	81
Du R	Eur Respir J	Hubei, China	From December 25, 2019 to February 7, 2020	March 24, 2020	179
Feng Y	Am J Respir Crit Care Med	3 provinces, China	From January 1, 2020 to February 15, 2020	March 21, 2020	476
Goyal P	N Eng J Med	New York, US	From March 5, 2020 to March 27 2020	April 10, 2020	393
Grasselli G	JAMA	Lombardia, Italy	From February 20, 2020 to March 18, 2020	March 25, 2020	1591
Grein J	N Eng J Med	Canada, Europe, Japan and US	From January 25, 2020 to March 7, 2020	March 7, 2020	53
Guan W	Eur Respir J	31 provinces, China	From December 11, 2020 to January 31, 2020	January 31, 2020	1590
Guo T	JAMA Cardiol	Hubei, China	From January 23, 2020 to February 23, 2020	February 23, 2020	187
Guo W	Diabetes Metab Res Rev	Hubei, China	From February 10, 2020 to February 29, 2020	March 3, 2020	174
Han Y	J Med Virol	Shaanxi, China	From January 31, 2020 to February 16, 2020	February 16, 2020	25^a^
He Y	Infect Control Hosp Epidemiol	Hubei, China	From December 30, 2019 to February 29, 2020	February 29, 2020	65
Huang C	Lancet	Hubei, China	From December 16, 2019 to January 2, 2020	January 22, 2020	41
Jin X	Gut	Zhejiang, China	From January 17, 2020 to February 8, 2020	February 9, 2020	74
Li J	JAMA Cardiol	Hubei, China	From January 15, 2020 to March 15, 2020	March 15, 2020	1178
Li R	J Clin Virol	Hubei, China	From January 20, 2020 to February 14, 2020	February 29, 2020	225
Liu K	J Infect	Hainan**,** China	From January 15, 2020 to February 18, 2020	February 18, 2020	56
Liu K	Chin Med J	9 tertiary hospitals in Hubei, China	From December 30, 2019 to January 24, 2020	January 24, 2020	137
Liu W	Chin Med J	Hubei, China	From December 30, 2020 to January 15, 2020	January 15, 2020	78
Liu Y	Platelets	Hubei, China	From January 2, 2020 to March 1, 2020	March 1, 2020	383
McMichael TM	N Eng J Med	Washington State, US	From February 27, 2020 to March 18, 2020	March 18, 2020	101^b^
Myers L	JAMA	California, US	From March 1, 2020 to March 31, 2020	April 9, 2020	377
Richardson S	JAMA	New York, US	From March 1, 2020 to April 4, 2020	April 4, 2020	5700
Shi H	Lancet Infect Dis	Hubei, China	From December 20, 2019 to January 23, 2020	February 8, 2020	81
Shi S	JAMA Cardiol	Hubei, China	From January 20, 2020 to February 10, 2020	February 15, 2020	416
Simonnet A	Obesity (Silver Spring)	Hauts-de-France, France	From February 27, 2020 to April 5, 2020	April 6, 2020	124
Tan C	J Med Virol	Hunan, China	From January 18, 2020 to February 10, 2020	February 20, 2020	27
Tang N	J Thromb Haemost	Hubei, China	From January 1, 2020 to February 13, 2020	March 13, 2020	449
Wang L	J Infect	Hubei, China	From January 1, 2020 to February 6, 2020	March 5, 2020	339
Wang Z	Clin Infect Dis	Hubei, China	From January 16, 2020 to January 29, 2020	February 4, 2020	69
Wu C	JAMA Intern Med	Hubei, China	From December 25, 2019 to January 26, 2020	February 13, 2020	201
Xu B	J Infect	Hubei, China	From December 26, 2019 to March 1, 2020	March 5, 2020	187
Yuan M	PLoS One	Hubei, China	From January 1, 2020 to January 25, 2020	January 25, 2020	27
Zha L	Med J Aust	Anhui, China	From January 24, 2020 to February 24, 2020	February 29, 2020	31
Zhang J	Allergy	Hubei, China	From December 29, 2020 to February 16, 2020	February 28, 2020	290
Zhang L	J Thromb Haemost	Hubei, China	From January 12, 2020 to March 15, 2020	March 15, 2020	343
Zhang P	Circ Res	Hubei, China	From December 31, 2019 to February 20, 2020	March 7, 2020	1128
Zhou F	Lancet	Hubei, China	From December 29, 2019 to January 31, 2020	January 31, 2020	191
Zhou Y	Clin Transl Sci	Hubei, China	From January 28, 2020 to March 02, 2020	March 02, 2020	21

*US* United States

^a^Only adult patients were considered in the present meta-analysis

^b^This study was conducted in a nursing facility. Only residents infected by SARS-CoV-2 were included in the present meta-analysis

**Fig. 2:**
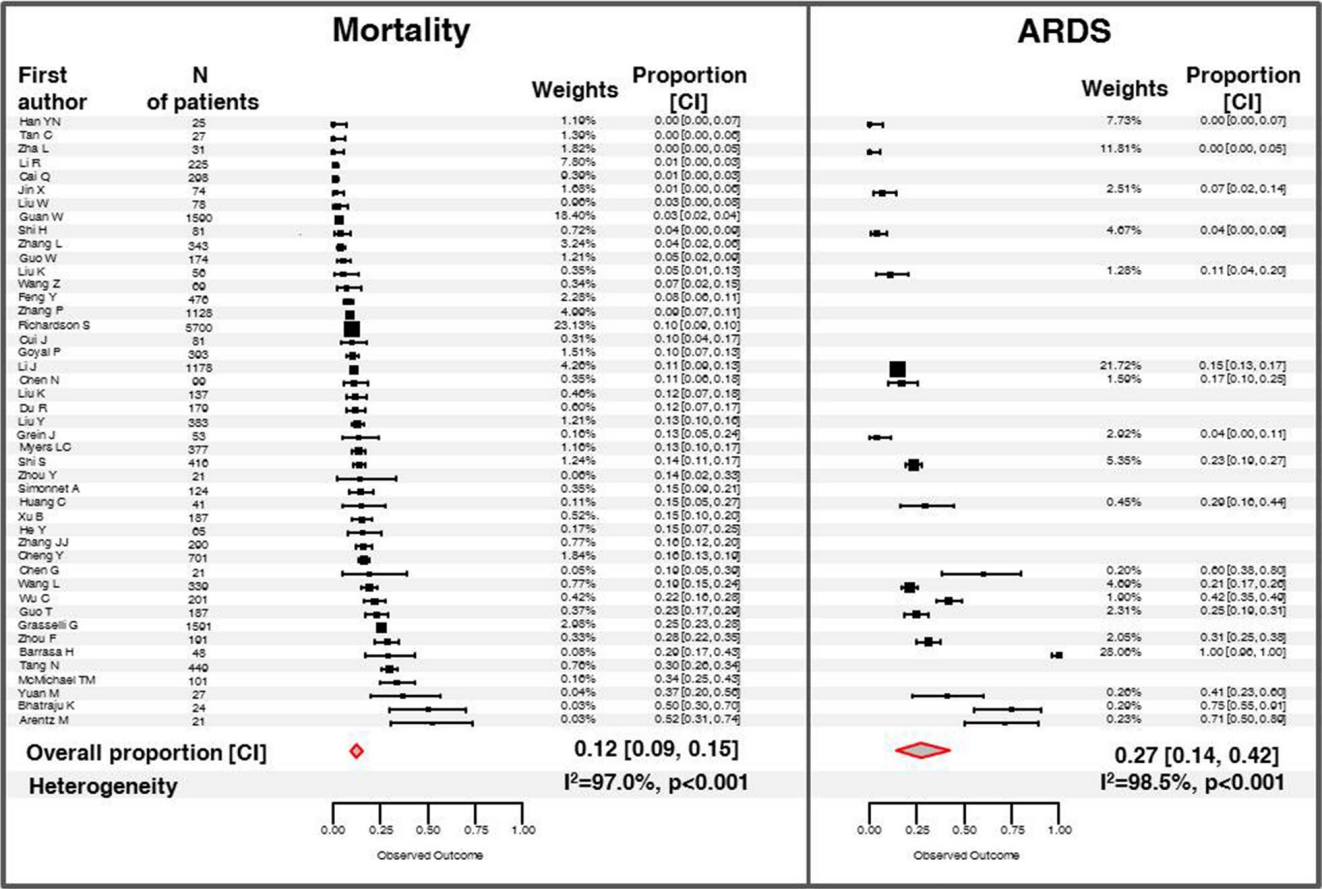
Individual and overall incidence for in-hospital mortality and ARDS. Solid squares indicate the weighted estimate of incidence for each single study; horizontal bars indicate 95% CI; red diamond indicates the overall estimated incidence

**Fig. 3:**
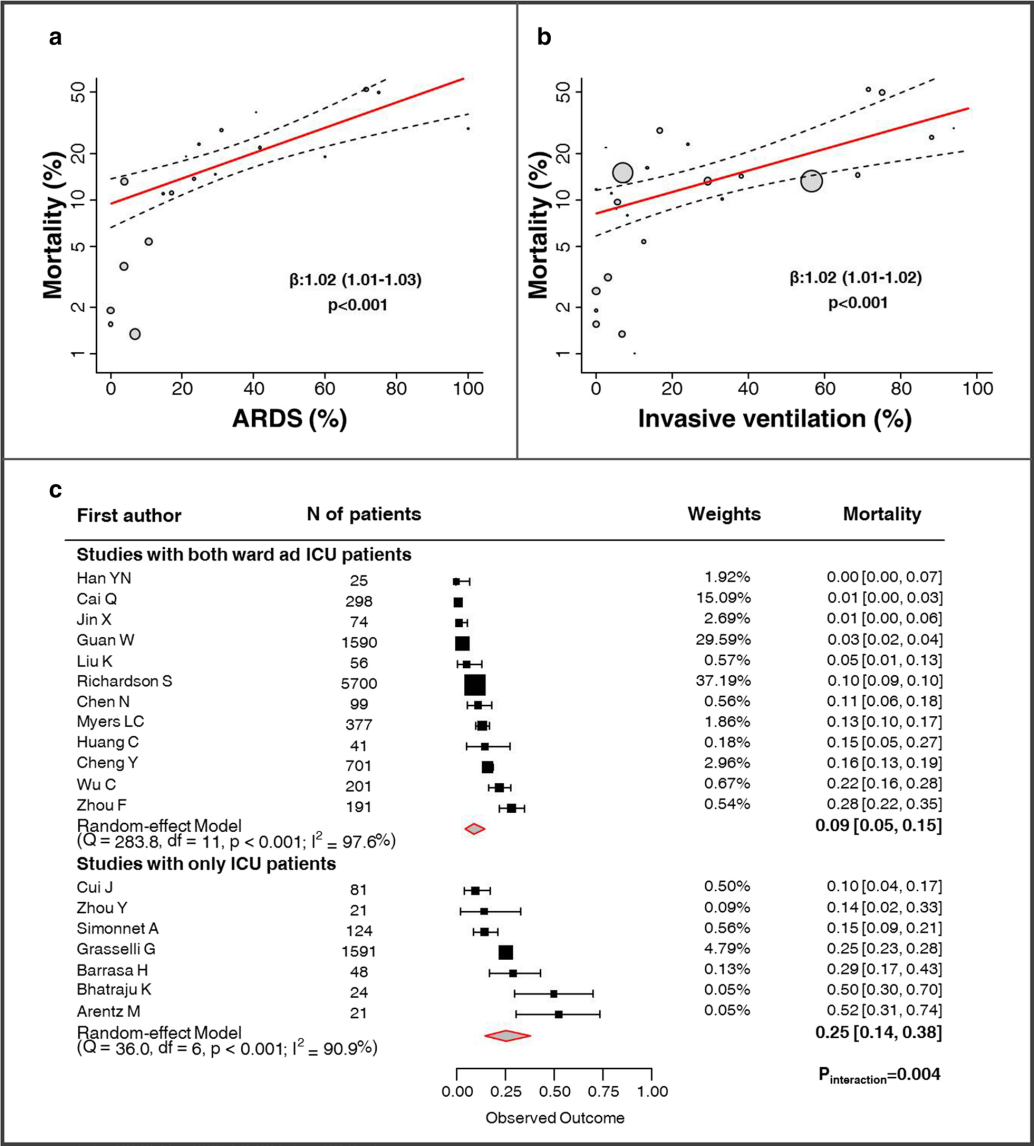
Exploratory analyses for the effects of ARDS, IMV and ICU on in-hospital mortality. Scatter plots showing the association between the proportion of ARDS and IMV use and in-hospital mortality (**a**,** b**). Each circle size represents a study, telescoped by its weight in the analysis. The x-axis shows the prevalence of each covariate. The y-axis shows the incidence of in-hospital mortality. The regression line is calculated by the univariable meta-regression model. Subgroup analysis for studies including only ICU patients versus studies enrolling mixed population (both ward and ICU; **c**). Solid squares indicate the weighted estimate of incidence for each single study; horizontal bars indicate 95% CI; red diamond indicates the overall estimated incidence. *ARDS* acute respiratory distress syndrome, *ICU* intensive care unit, *IMV* invasive mechanical ventilation

The pooled estimate of in-hospital mortality was 12% (95% CI 9–15%) although affected by a high heterogeneity degree among studies (I^2^: 96.5%; p < 0.001; Fig. 2). ARDS was reported in 27% of cases (95% CI 14–42%; Fig. 2) and significantly correlated with the rate of death across reports (coefficient: 1.02; 95% CI 1.01–1.03; p < 0.001; Fig. 3). Moreover, the proportion of IMV use, another index of COVID-19 severity, was significantly higher among studies reporting a higher incidence of in-hospital mortality (coefficient: 1.02; 95% CI 1.01–1.02; p < 0.001; Fig. 3).

Data on clinical setting were available in 19 studies (Fig. 3). Seven studies were conducted exclusively on ICU patients; the remaining studies enrolled mixed populations (both ICU and ward) with a mean proportion of patients admitted in ICU of 14.6% (95% CI 9.0–21.2%). The risk of in-hospital death was significantly higher in the studies restricted to the ICU setting (25%, 95% CI 14–38%) as compared to the mixed cohorts (9%, 95% CI 5–15%; P_interaction_ = 0.004).

**Fig. 4:**
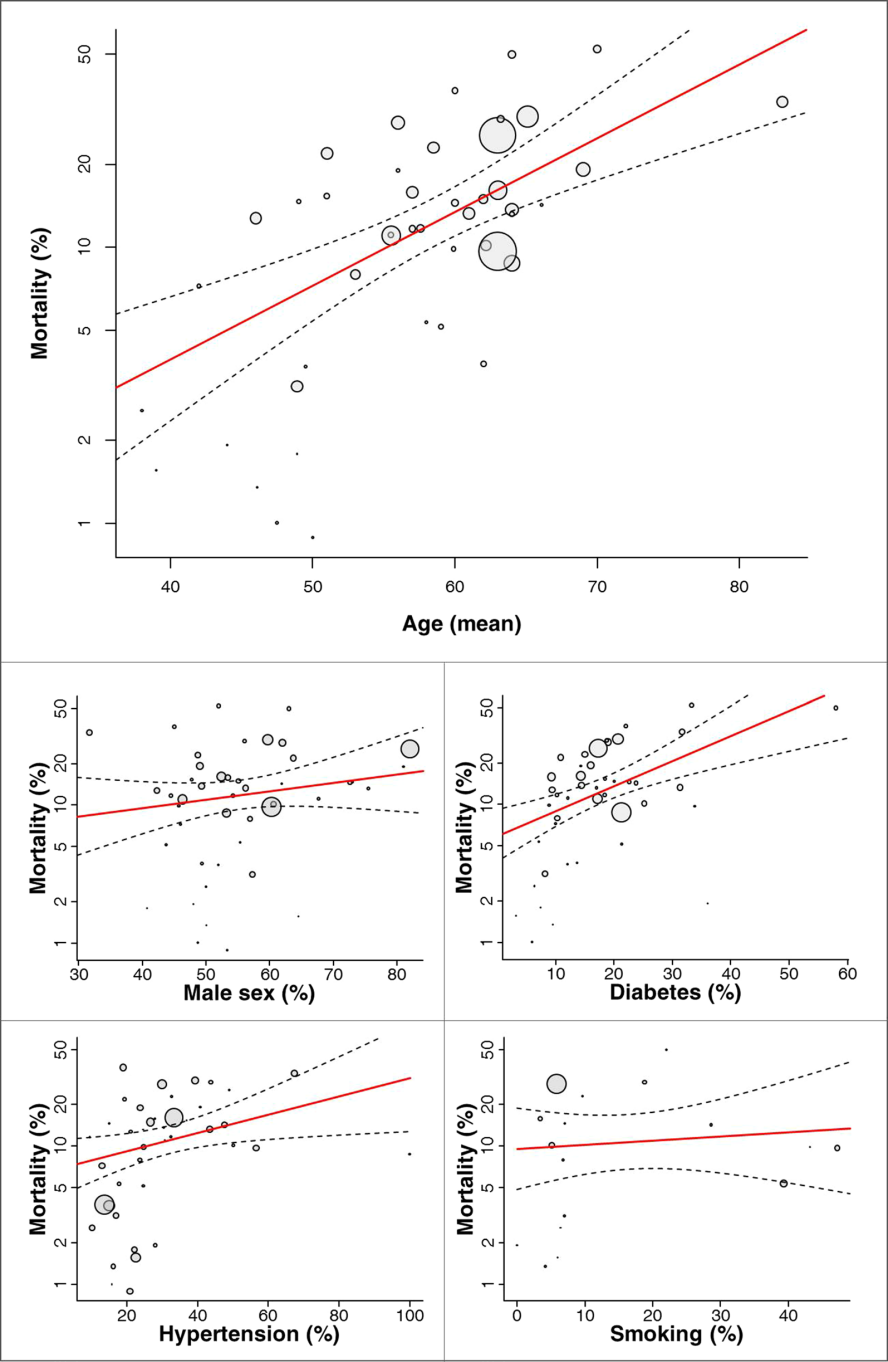
Meta-Regression analysis of the effects of cardiovascular risk factors on in-hospital mortality. Scatter plots showing the association between cardiovascular risk factors and in-hospital mortality. Each circle size represents a study, telescoped by its weight in the analysis. The x-axis shows the prevalence of each covariate. The y-axis shows the incidence of in-hospital mortality. The regression line is calculated by the univariable meta-regression model

The univariable meta-regression analyses between cardiovascular risk factors and in-hospital mortality are displayed in Fig. 4 and Table 2. Age was available in all the studies and resulted significantly associated with in-hospital mortality (coefficient: 1.06; 95% CI 1.04–1.09; p < 0.001). Diabetes was reported in 43 of 45 studies and resulted significantly associated with the study outcome (coefficient: 1.04; 95% CI 1.02–1.07; p < 0.001). Hypertension was available in 42 of 45 studies and significantly correlated with in-hospital mortality (coefficient: 1.01; 95% CI 1.01–1.03; p = 0.013). Male sex (coefficient: 1.01; 95% CI 0.99–1.03; p = 0.197) and smoking (coefficient: 1.01; 95% CI 0.98–1.04; p = 0.653) did not show significant associations with fatal outcome.

**Table 2:** Univariable and multivariable meta-regression analyses of the effects of cardiovascular risk factors on in-hospital mortality

Univariable analysis				Multivariable analysis		
Variable	Coefficient	[CI]	*p* value	Variables	Coefficient	[CI]
Age	1.061	[1.037, 1.086]	< 0.001	**–**	**–**	**–**
				–	**–**	**–**
Male sex	1.012	[0.994, 1.031]	0.1967	Male sex	1.015	[0.998, 1.033]
				Age	1.063	[1.039, 1.088]
Hypertension	1.014	[1.003, 1.025]	0.0133	Hypertension	0.999	[0.986, 1.011]
				Age	1.056	[1.025, 1.087]
Diabetes	1.043	[1.021, 1.065]	< 0.001	Diabetes	1.024	[1.001, 1.047]
				Age	1.042	[1.013, 1.071]
Smoking	1.007	[0.977, 1.036]	0.6534	Smoking	0.977	[0.950, 1.004]
				Age	1.120	[1.056, 1.189]

*CI* confidence interval

At multivariable age-adjusted meta-regression analysis, diabetes was significantly associated with in-hospital mortality (coefficient: 1.02; 95% CI 1.01–1.05; p = 0.043); conversely, hypertension was no longer significant after adjustment for age (coefficient: 1.00; 95% CI 0.99–1.01; p = 0.820; Table 2). A significant association between age and the study outcome was confirmed in each of the multivariable models.

### Quality assessment and publication bias

Quality assessment is detailed in Additional file 2. All the included studies showed an adequate-to-good quality. Thirty-two of 45 studies showed scores ≥ 6 according to the Newcastle–Ottawa scale; a score of 5 was observed in the remaining 13.

**Fig. 5:**
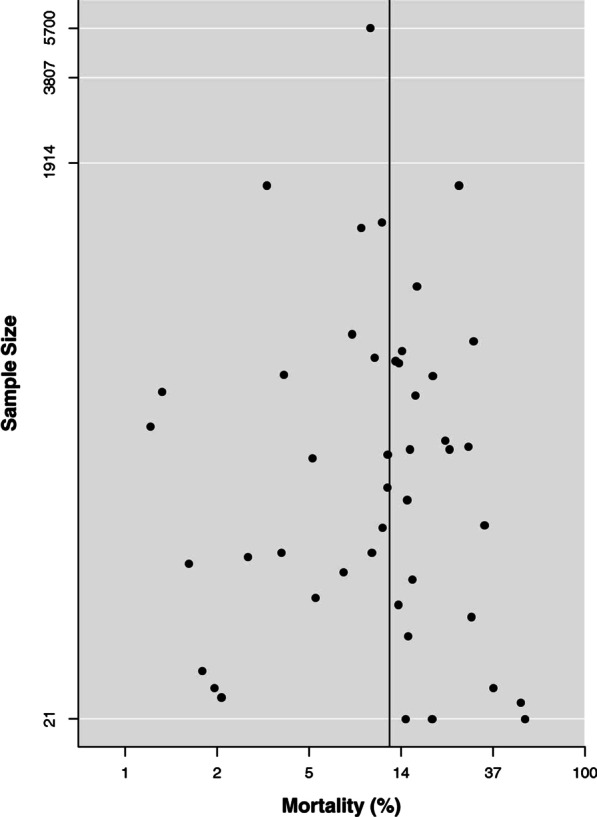
Funnel plot for in-hospital mortality. The analysis showed no asymmetry suggestive for a significant risk of publication bias

Visual inspection of funnel plots and the rank correlation test showed no significant asymmetry (Kendall's tau = -0.575, p = 0.565), suggesting that in-hospital mortality did not depend on the size of the studies (Fig. 5).

## Discussion

The search for potential associations between patient characteristics and outcome may be helpful to stratify prognosis, to plan patient clinical management and to optimize sources in COVID-19 outbreak. Although many cardiovascular risk factors have been associated with adverse outcome, confounding and residual confounding could lead to apparent associations that might not represent genuine effects or can bias the magnitudes of effects. This hazard is higher in small series, single-centre (and single-country) studies, and selective reports from special populations. [55].

By including 45 studies and 18,300 patients, this is the largest meta-analysis investigating the effect of cardiovascular risk factors on in-hospital mortality in COVID-19, by using crude and multivariable meta-regression models, available so far. The main findings of this study can be summarized as follows: (I) COVID-19 was associated to a high risk of in-hospital death, which occurred in about one patient in 8; (II) studies with high percentage of ARDS and IMV use, two indexes of disease severity, as well as of ICU admission, reported the highest risk of death during hospitalization; (III) age and diabetes were independent predictors of mortality; (IV) after adjustment for age, hypertension did not show any significant association with the risk of in-hospital death.

The mortality estimate of COVID-19 in the general population depends on the number of deaths relative to the number of confirmed cases of infection, which is not representative of the actual death rate, and may be substantially overestimated. [56, 57] Real-world studies conducted on inpatient populations have the advantage to provide the real proportion of mortality among subjects with confirmed COVID-19 diagnosis. The overall incidence of death detected in this analysis was clearly high, emphasizing the hazard of COVID-19 in patients who required hospitalization. These results were consistent with a recent meta-analysis by Sabatino et al., which included 21 studies reporting clinical data on hospitalized patients infected by SARS-CoV-2. They found a fatality rate of 9.6%, with large heterogeneity among studies, and reported a significant association of age and pre-existing cardiovascular risk factors with the risk of in-hospital mortality at univariable meta-regression analysis. [58].

The pooled mortality estimate in our analysis was affected by a wide inconsistency between patient cohorts. There are many reasons for the effect variability detected across observational studies including clinical setting (ICU, ward), the prevalence of in-hospital complications (e.g. ARDS) and the need of invasive mechanical supportive measures, which may play as effect modifiers. We tried to account for this variability by performing exploratory analyses of the relationships between the rate of mortality and the proportion of ARDS and IMV use, which are indexes of disease severity. As expected, the higher the percentage of ARDS/IMV among patients enrolled, themm higher was the rate of death during in-hospital course. In addition, the subgroup including only studies conducted in ICU, compared to the mixed group, showed a significantly higher risk of death. These analyses were still affected by high residual heterogeneity suggesting that these variables, although able to stratify prognosis, could not account for the differences among the studies included in this meta-analysis. Our analysis could not account for multiple variables as well as for concealed confounders such as the availability of health-care resources, in-hospital paths, healthcare systems and strategies adopted in the context of pandemic between centers or countries. [4] Actually, high inconsistency is a frequent finding in meta-analyses of observational studies and should be weighed against inference statistics considerations. If the goal of a meta-analysis is to evaluate the direction of a possible association, as in our study, its results can be acceptable despite a certain inconsistency among studies. [59].

The association of age with mortality is an expected finding; noteworthy, this significant correlation persisted in multiple adjusted meta-regression models including other cardiovascular risk factors. This result underlies the importance of individual preventive and protective measures during COVID-19 public health crisis and of dedicated healthcare strategies for elderly patients during hospitalization.

Diabetes is the second most common comorbidity (after hypertension) in hospitalized patients with COVID-19, and its prevalence increase with age. [7, 26, 60] Our analysis showed that diabetic patients infected by SARS-CoV-2, as compared to those without diabetes, have an higher risk for in-hospital death independently from age. Beyond the inherent association of diabetes with atherosclerosis and cardiovascular disease, bad glycemic control may negatively affect the outcome of patients with diabetes infected by SARS-CoV-2 through different mechanisms [61]: corticosteroid therapy, high glucose level related to septic status, inadequate glucose monitoring in patients with infection, lack of contact with healthcare professional qualified on diabetes management, and angiotensin-converting enzyme inhibitors (ACEi) withdrawal.

SARS-CoV-2 binds to the zinc peptidase angiotensin-converting enzyme 2 (ACE2), a surface molecule expressed by endothelial cells of arteries and veins, arterial smooth muscle, epithelial cells, and immune cells. [62] Some authors hypothesized a relationship between diabetes and virulence of SARS-CoV-2 infection, since hyperglycemia might favor virus entrance into immune cells by increasing the expression of ACE2.

Our study demonstrates that the association between hypertension and mortality in hospitalized patients with COVID-19 reflect largely the older age of these patients. In fact, the significant correlation observed at the univariable meta-regression model was not confirmed after adjustment for age. Preliminary studies suggested that hypertension was a risk factor for in-hospital outcome in patients with COVID-19. [7, 10, 26, 60] Based on these findings, some authors hypothesized that ACEi or angiotensin II receptor blockers (ARBs) might increase the expression of ACE2 in animal models and facilitate virus entry into the host cells. [63] However, these studies reported only descriptive or univariable regression analyses, not accounting for potential confounders, and did not demonstrate an independent association between hypertension and fatal outcome. Actually, a recent multi-center propensity-score matching study on 1,128 COVID-19 patients with hypertension showed a significantly lower risk of 28-day all-cause mortality and of septic shock in ACEi/ARBs group versus non-ACEi/ARBs [52]. This finding was confirmed in a recent study on 8,910 from 169 hospital, which showed that no risk of in-hospital death was associated with the use of ACEi/ARBs at multivariable analysis [64].

The association between smoking and mortality in COVID-19, though plausible, is still debated [65, 66]. A recent study by Williamson and colleagues on 17,278,392 adults from the OpenSAFELY platform, showed that current smokers were also associated with a lower risk of mortality related to COVID-19 [HR: 0.89 (0.82–0.97)] compared to never smokers [67]. In that analysis, the hazard ratio could not be interpreted causally owing to the inclusion of factors that were likely to mediate smoking effects (e.g. the association was no longer significant after adjustment for COPD). Thus, whether smoking is associated with higher risk of mortality need to be clarified in future studies as the epidemic progresses and more data accumulate.

The recent onset and the variable dissemination pattern of the outbreak make difficult to draw firm conclusions on the clinical characteristics and epidemiology of COVID-19. Our data indicate that elderly and diabetic patients are higher-risk populations, and suggest taking utmost care in clinical management of these patients during hospitalization.

Continuous surveillance, with reporting of patient characteristics worldwide, are required to confirm our findings and more deeply understand the relationship between cardiovascular risk factors and outcome of patients infected by SARS-CoV-2.

## Study limitations

Some limitations of our study should be acknowledged. Although we included 45 studies enrolling 18,300 patients, results of meta-analyses are hypothesis-generating and should be interpreted accordingly.

The observational nature of the studies included might have contributed to the heterogeneity observed in this analysis. We tried to manage this issue by performing univariable meta-regressions for ARDS and IMV as well as a subgroup analysis for ICU vs ICU/ward. Although these analyses showed significant and clinically valuable results, they were still affected by a substantial residual heterogeneity.

Owing to the intrinsic limitations of study-level analyses, we could not account for multiple potential confounders and we adjusted only for age, considered the most important confounding factor in this clinical setting. Moreover, we could not adjust for hidden confounders as well as for patient characteristics non-systematically or rarely reported.

Many authors reported incomplete clinical and instrumental information (i.e. prevalence of coronary heart disease, heart failure, chronic kidney disease, left ventricular ejection fraction, etc.) due to factors such as critically ill patients, operator’s fear of contagion, and in-hospital paths. We may hypothesize that difficulties in data collection/storage and limited feasibility/repeatability of instrumental exams may have affected the completion and granularity of data.

To meet the urgent need of clinical reports on COVID-19, many studies encompassed a relatively short follow-up time as compared to the course of the disease, and some of them ended before the discharge or death of each patients enrolled. Although we tried to manage this issue by excluding the studies with higher number of open cases at the end, a certain underestimation of mortality is likely. We do not claim to provide the exact estimate of in-hospital mortality in COVID-19 pandemic, but to explore the potential association between cardiovascular risk factors and outcome. Since the number of pandemic-focused studies is increasing, and clinical observation of these patients is ongoing, the current mortality pattern might change to some extent in the next weeks or months.

The majority of studies included in this analysis reported data from Chinese populations, and only ten reports were from other countries (3 from Europe and 7 from US). This issue might have affected the generalizability of our findings, and requires confirmation by large worldwide studies.

Among the cardiovascular risk factors, we did not analyze unfrequently reported conditions such as obesity, dyslipidemia, and family history of cardiovascular disease. Indeed, these data were seldom reported in the items selected for this meta-analysis, and were not sufficient for inference analysis.

## Conclusions

This study suggests that older age and diabetes are associated to higher risk of in-hospital mortality in patients infected by SARS-CoV-2. Conversely, hypertension, male sex and smoking did not independently correlate with fatal outcome at multivariable meta-regression analysis.

Although difficult to realize in a pandemic scenario, large multicenter cross-national studies are warranted to improve our understanding on the association between cardiovascular risk factors and mortality in COVID-19.

## Supplementary Information

**Additional file 1:** Main characteristics of the studies included in the analysis.

**Additional file 2:** Quality assessment of the studies based on the Newcastle–Ottawa scale.

## Data Availability

All data generated or analyzed during this study are included in this published article (and its supplementary information files).

## Abbreviations

ACE2: Angiotensin-converting enzyme 2
ACEi: Angiotensin-converting enzyme inhibitors
ARBs: Angiotensin II receptor blockers
ARDS: Acute respiratory distress syndrome
CI: Confidence interval
COVID-19: Coronavirus disease 2019
ICU: Intensive care unit
IMV: Invasive mechanical ventilation
MOOSE: Meta-analysis of observational studies in epidemiology statement
SARS-CoV-2: Severe acute respiratory syndrome coronavirus 2
