# Supplementary material for: Cardiovascular risk factors and mortality in hospitalized patients with COVID-19: systematic review and meta-analysis of 45 studies and 18,300 patients
Source: BMC Cardiovasc Disord. 2021 Jan 7;21:23. doi: 10.1186/s12872-020-01816-3 (PMC7789083; doi:10.1186/s12872-020-01816-3)
Supplement: Supplementary file 1 — Additional file 1: Main characteristics of the studies included in the analysis. [file 12872_2020_1816_MOESM1_ESM.docx]

**Additional file 1. Main characteristics of the studies included in the analysis.**

| **Author** | **Journal** | **Patients, N** | **Age, years** | **Males, %** | **HT, %** | **DM, %** | **Smoke, %** | **ICU, %** | **ARDS, %** | **IMV, %** |
| --- | --- | --- | --- | --- | --- | --- | --- | --- | --- | --- |
| Arentz M | JAMA | 21 | 70 | 52 | NA | 33.3 | NA | 100 | 71.4 | 71.4 |
| Barrasa H | Am J Respir Crit Care Med | 48 | 63.2 | 56 | 43.8 | 18.8 | 18.8 | 100 | 100 | 93.8 |
| Bhatraju K | N Eng J Med | 24 | 64 | 63 | NA | 58 | 22 | 100 | 75 | 75 |
| Cai Q | Allergy | 298 | 47.5 | 48.7 | 15.8 | 6 | NA | 10.1 | NA | 10.1 |
| Chen G | J Clin Invest | 21 | 56 | 81 | 23.8 | 14.3 | NA | NA | 60 | NA |
| Chen N | Lancet | 99 | 55.5 | 67.7 | NA | 12.1 | NA | 23.2 | 17.1 | 4 |
| Cheng Y | Kidney Int | 701 | 63 | 52.4 | 33.4 | 14.3 | NA | 10.4 | NA | 13.4 |
| Cui J | J Thromb Haemost | 81 | 59.9 | 46 | 25 | 10 | 43 | 100 | NA | NA |
| Du R | Eur Respir J | 179 | 57.6 | 54.2 | 32.4 | 18.4 | NA | NA | NA | NA |
| Feng Y | Am J Respir Crit Care Med | 476 | 53 | 56.9 | 23.7 | 10.3 | 9.7 | NA | NA | 8.2 |
| Goyal P | N Eng J Med | 393 | 62.2 | 60.6 | 50.1 | 25.2 | 5.1 | NA | NA | 33.1 |
| Grasselli G | JAMA | 1591 | 63 | 82 | 49 | 17.3 | NA | 100 | NA | 88 |
| Grein J | N Eng J Med | 53 | 64 | 75 | 25 | 17 | NA | NA | 3.8 | 56.6 |
| Guan W | Eur Respir J | 1590 | 48.9 | 57.3 | 16.9 | 8.2 | 7 | 6.2 | NA | 3.1 |
| Guo T | JAMA Cardiol | 187 | 58.5 | 48.7 | 32.6 | 15 | 9.6 | NA | 24.6 | 24.1 |
| Guo W | Diabetes Metab Res Rev | 174 | 59 | 43.7 | 24.7 | 21.3 | NA | NA | NA | NA |
| Han Y | J Med Virol | 25^*^ | 44 | 48 | 28 | 36 | 0 | 0 | 0 | 0 |
| He Y | Infect Control Hosp Epidemiol | 65 | 51 | 47.7 | 36.9 | 18.5 | NA | NA | NA | NA |
| Huang C | Lancet | 41 | 49 | 73 | 15 | 20 | 7 | 31.7 | 29.2 | 5 |
| Jin X | Gut | 74 | 46.1 | 50 | 16.2 | 9.5 | 4.2 | 6.8 | 6.8 | 6.8 |
| Li J | JAMA Cardiol | 1178 | 55.5 | 46.3 | 30.7 | 17.2 | NA | NA | 14.6 | NA |
| Li R | J Clin Virol | 225 | 50 | 53.3 | 20.9 | NA | NA | NA | NA | NA |
| Liu K | J Infect | 56 | 58 | 55.3 | 17.8 | 7.1 | 39.3 | 16 | 10.7 | 12.5 |
| Liu K | Chin Med J | 137 | 57 | 44.5 | 9.5 | 10.2 | 1.5 | NA | NA | 0 |
| Liu W | Chin Med J | 78 | 38 | 50 | 10.2 | 6.4 | 6.4 | NA | NA | 0 |
| Liu Y | Platelets | 383 | 46 | 42.3 | 21.1 | 9.4 | NA | NA | NA | NA |
| McMichael TM | N Eng J Med | 101^*^ | 83 | 31.7 | 67.3 | 31.7 | NA | NA | NA | NA |
| Myers L | JAMA | 377 | 61 | 56.2 | 43.5 | 31.3 | NA | 30 | NA | 29.2 |
| Richardson S | JAMA | 5700 | 63 | 60.3 | 56.6 | 33.8 | 47.2 | 6.5 | NA | 5.6 |
| Shi H | Lancet Infect Dis | 81 | 49.5 | 51.9 | 15 | 12 | NA | NA | 3.7 | NA |
| Shi S | JAMA Cardiol | 416 | 64 | 49.3 | 30.5 | 14.4 | NA | NA | 23.3 | 7.7 |
| Simonnet A | Obesity (Silver Spring) | 124 | 60 | 72.6 | 48.4 | 22.6 | NA | 100 | NA | 68.6 |
| Tan C | J Med Virol | 27 | 48.9 | 40.7 | 22.2 | 7.4 | NA | NA | NA | NA |
| Tang N | J Thromb Haemost | 449 | 65.1 | 59.7 | 39.4 | 20.7 | NA | NA | NA | NA |
| Wang L | J Infect | 339 | 69 | 49 | 40.8 | 16 | NA | NA | 21 | NA |
| Wang Z | Clin Infect Dis | 69 | 42 | 46 | 13 | 10 | NA | NA | NA | NA |
| Wu C | JAMA Intern Med | 201 | 51 | 63.7 | 19.4 | 10.9 | NA | 26.4 | 41.8 | 2.5 |
| Xu B | J Infect | 187 | 62 | 55.1 | 26.7 | NA | NA | NA | NA | 7 |
| Yuan M | PLoS One | 27 | 60 | 45 | 19 | 22 | NA | NA | 40.7 | NA |
| Zha L | Med J Aust | 31 | 39 | 64 | 22.6 | 3.2 | 6 | NA | 0 | 0 |
| Zhang J | Allergy | 290 | 57 | 53.4 | 27.9 | 9.3 | 3.4 | NA | NA | NA |
| Zhang L | J Thromb Haemost | 343 | 62 | 49.3 | 22.1 | 13.7 | NA | NA | NA | NA |
| Zhang P | Circ Res | 1128 | 64 | 53.2 | 100 | 21.3 | NA | NA | NA | 5.3 |
| Zhou F | Lancet | 191 | 56 | 62 | 30 | 19 | 6 | 26 | 30.9 | 16.7 |
| Zhou Y | Clin Transl Sci | 21 | 66.1 | 61.9 | 47.6 | 23.8 | 28.6 | 100 | NA | 38.1 |

ARDS, acute respiratory distress syndrome; DM, Diabetes Mellitus; HT, Hypertension; ICU, intensive care unit; IMV, invasive mechanical ventilation; NA, not available;

*This study was conducted in a nursing facility. Only residents infected by SARS-CoV-2 were included in the present meta-analysis.
