# Supplementary material for: Cardiovascular risk factors and mortality in hospitalized patients with COVID-19: systematic review and meta-analysis of 45 studies and 18,300 patients
Source: BMC Cardiovasc Disord. 2021 Jan 7;21:23. doi: 10.1186/s12872-020-01816-3 (PMC7789083; doi:10.1186/s12872-020-01816-3)
Supplement: Supplementary file 2 — Additional file 2: Quality assessment of the studies based on the Newcastle–Ottawa scale. [file 12872_2020_1816_MOESM2_ESM.docx]

| **Additional file 2. Quality assessment of the studies based on the Newcastle-Ottawa scale.** | | | | | | | | | | |
| --- | --- | --- | --- | --- | --- | --- | --- | --- | --- | --- |
|  | **SELECTION** | | | | **COMPARABILITY** | | **OUTCOME** | | | TOTAL |
|  | Representativeness of the cohort study | Selection of the non-exposed cohort | Ascertainment of exposure | Outcome not present at start of study | Control of main factor | Control for additional factor | Outcome assessment | Length of FU | Adequacy of FU |  |
| Arentz M | * | 0 | * | * | 0 | 0 | * | * | 0 | 5 |
| Barrasa H | * | 0 | * | * | 0 | 0 | * | * | 0 | 5 |
| Bhatraju K | * | 0 | * | * | 0 | 0 | * | * | 0 | 5 |
| Cai Q | * | 0 | * | * | * | * | * | * | * | 8 |
| Chen G | * | 0 | * | * | 0 | 0 | * | * | * | 5 |
| Chen N | * | 0 | * | * | 0 | 0 | * | * | * | 6 |
| Cheng Y | * | * | * | * | * | * | * | * | 0 | 8 |
| Cui J | * | 0 | * | * | 0 | 0 | * | * | * | 6 |
| Du R | * | 0 | * | * | * | * | * | * | * | 8 |
| Feng Y | * | 0 | * | * | * | * | * | * | 0 | 7 |
| Goyal P | * | 0 | * | * | 0 | * | * | * | 0 | 6 |
| Grasselli G | * | 0 | * | * | * | * | * | * | 0 | 7 |
| Grein J | * | 0 | * | * | * | * | * | * | 0 | 7 |
| Guan W | * | * | * | * | * | * | * | 0 | 0 | 7 |
| Guo T | * | * | * | * | * | 0 | * | * | * | 8 |
| Guo W | * | * | * | * | * | 0 | * | 0 | 0 | 6 |
| Han Y | * | * | * | * | 0 | 0 | * | * | * | 7 |
| He Y | 0 | 0 | * | * | 0 | 0 | * | * | * | 5 |
| Huang C | * | 0 | * | * | 0 | 0 | * | * | 0 | 5 |
| Jin X | * | * | * | * | * | * | * | 0 | 0 | 7 |
| Li R | * | 0 | * | * | 0 | 0 | * | * | 0 | 5 |
| Li J | * | * | * | * | * | * | * | * | 0 | 8 |
| Liu K | * | * | * | * | 0 | 0 | * | * | * | 7 |
| Liu K | * | 0 | * | * | 0 | 0 | * | * | 0 | 5 |
| Liu W | * | 0 | * | * | 0 | 0 | * | 0 | * | 5 |
| Liu Y | * | * | * | * | 0 | * | * | * | 0 | 7 |
| McMichael TM | * | 0 | * | * | 0 | 0 | * | * | 0 | 5 |
| Myers LC | * | 0 | * | * | 0 | 0 | * | * | * | 6 |
| Richardson S | * | 0 | * | * | 0 | 0 | * | * | 0 | 5 |
| Shi H | * | 0 | * | * | * | 0 | * | * | 0 | 6 |
| Shi S | * | * | * | * | * | * | * | 0 | 0 | 7 |
| Simonnet A | * | 0 | * | * | * | * | * | * | 0 | 7 |
| Tan C | * | * | * | * | 0 | 0 | * | * | * | 7 |
| Tang N | * | 0 | * | * | * | * | * | * | 0 | 7 |
| Wang L | * | 0 | * | * | * | * | * | * | 0 | 7 |
| Wang Z | * | * | * | * | * | 0 | * | 0 | 0 | 6 |
| Wu C | * | 0 | * | * | * | * | * | * | * | 8 |
| Xu B | * | 0 | * | * | 0 | 0 | * | * | 0 | 5 |
| Yuan M | * | 0 | * | * | 0 | 0 | * | * | * | 6 |
| Zha L | * | * | * | * | * | * | * | * | 0 | 8 |
| Zhang JJ | * | * | * | * | * | * | * | * | 0 | 8 |
| Zhang L | * | * | * | * | 0 | 0 | * | * | 0 | 6 |
| Zhang P | * | * | * | * | * | * | * | * | * | 9 |
| Zhou F | * | 0 | * | * | * | * | * | * | * | 8 |
| Zhou Y | * | 0 | * | * | 0 | 0 | * | * | 0 | 5 |

FU, follow-up.
